# Characterization of the complete chloroplast genome of *Houttuynia cordata* Thunb and phylogenetic relationships

**DOI:** 10.1080/23802359.2019.1710604

**Published:** 2020-01-14

**Authors:** Xue-Lian Yang

**Affiliations:** College of Agriculture, Guizhou University, Guiyang, PR China

**Keywords:** *Houttuynia cordata* Thunb, chloroplast genome, Piperales, phylogenetic relationship analysis

## Abstract

In this study, I presented the chloroplast genome of *Houttuynia cordata* Thunb using BGISEQ-500 sequencing data. Its chloroplast genome is 160,226 bp in size. It contains a pair of inverted repeat regions of 26,853 bp, each separating a small single copy region of 18,340 bp and a large single copy region of 88,180 bp. Totally, 112 unique genes, including 78 protein coding genes, 30 tRNAs and 4 rRNAs, were identified and annotated in the chloroplast genome. Phylogenetic maximum likelihood analysis indicated that *H. cordata* Thunb is closest to *Piper cenocladu.*

*Houttuynia cordata* Thunb, belonging to Family Saururaceae of Piperales order, is a herbaceous plant with a fishy smell. The up-ground part of it is the source of Chinese herb medicine Herba houttuyniae, which is of great antibacterial and antiviral activities and diuretic and immunity-improving effects. It usually grows wild in damp places or near mountain streams in south China. It is also cultivated in some provinces of China such as Hunan province because its underground stem is a popular vegetable. In this study, I assembled and characterized its chloroplast genome using BGISEQ-500 sequencing data. Its relationship with several plant species of Piperales order was also investigated.

The specimen sample of *H. cordata* Thunb was isolated from Teaching Experiment Farm of Guizhou University, Guiyang, Guizhou Province, China (26°21′29″N; 106°41′01″E) and sample was deposited at Guizhou University. Total genomic DNA was extracted from fresh leaves according to my previous study (Yang et al. 2019), stored at the Guizhou University (No. YXC01) and was used for the shotgun library construction and BGISEQ-500 sequencing to generate about 1 Gbp data. The obtained high quality reads were firstly aligned to the chloroplast genomes of plant species from Piperales, including *Chloranthus erectus* (NC_039627.1), *Chloranthus japonicus* (KP256024.1), *Chloranthus spicatus* (EF380352.1), *Piper cenocladum* (DQ887677.1), *Piper kadsura* (NC_027941.1), *Piper laetispicum* (NC_042254.1) and *Sarcandra glabra* (MH939147.1). Chloroplast genome assembly and annotation was performed according to Zhang et al. (2019). The annotated chloroplast genome of *H. cordata* Thunb has been deposited in Genbank with the accession number MN541092.

The *H. cordata* Thunb chloroplast genome is 160,226 bp in size. It contains a pair of inverted repeat regions (26,853 bp), each separating a small single copy region of 18,340 bp and a large single copy region of 88,180 bp. Totally, 112 unique genes was annotated in the *H. cordata* chloroplast genome, including 78 protein coding genes, 30 tRNAs and four rRNAs. Among these genes, six protein coding genes (*ndhB*, *rpl2*, *rpl23*, *rps12*, *rps7* and *ycf2*), four rRNAs (*rrn4.5*, *rrn5*, *rrn16* and *rrn23*) and seven tRNAs (*trnA-UGC*, *trnI-CAU*, *trnI-GAU*, *trnL-CAA*, *trnN-GUU*, *trnR-ACG* and *trnV-GAC*) occurred in two copies. The overall nucleotide composition of the chloroplast genome is: 30.4% A, 31.2% T, 19.4% C, and 19.0% G, with the total GC content of 38.4%.

Sequence alignment analysis was performed using chloroplast genome sequences of three plant species belonging to Piperaceae, four plant species from Chloranthaceae and the *H. cordata* chloroplast genome using HomBlocks pipeline (Bi et al. 2018). Maximum likelihood tree was constructed according to the method described by Wu et al. (2019). Phylogenetic maximum likelihood analysis indicated that *H. cordata* Thunb is closest to *P. cenocladum* (Figure 1). The complete chloroplast genome of *H. cordata* Thunb would provide valuable genetic information for researches of plants belonging to the Piperales order.

**Figure 1.**
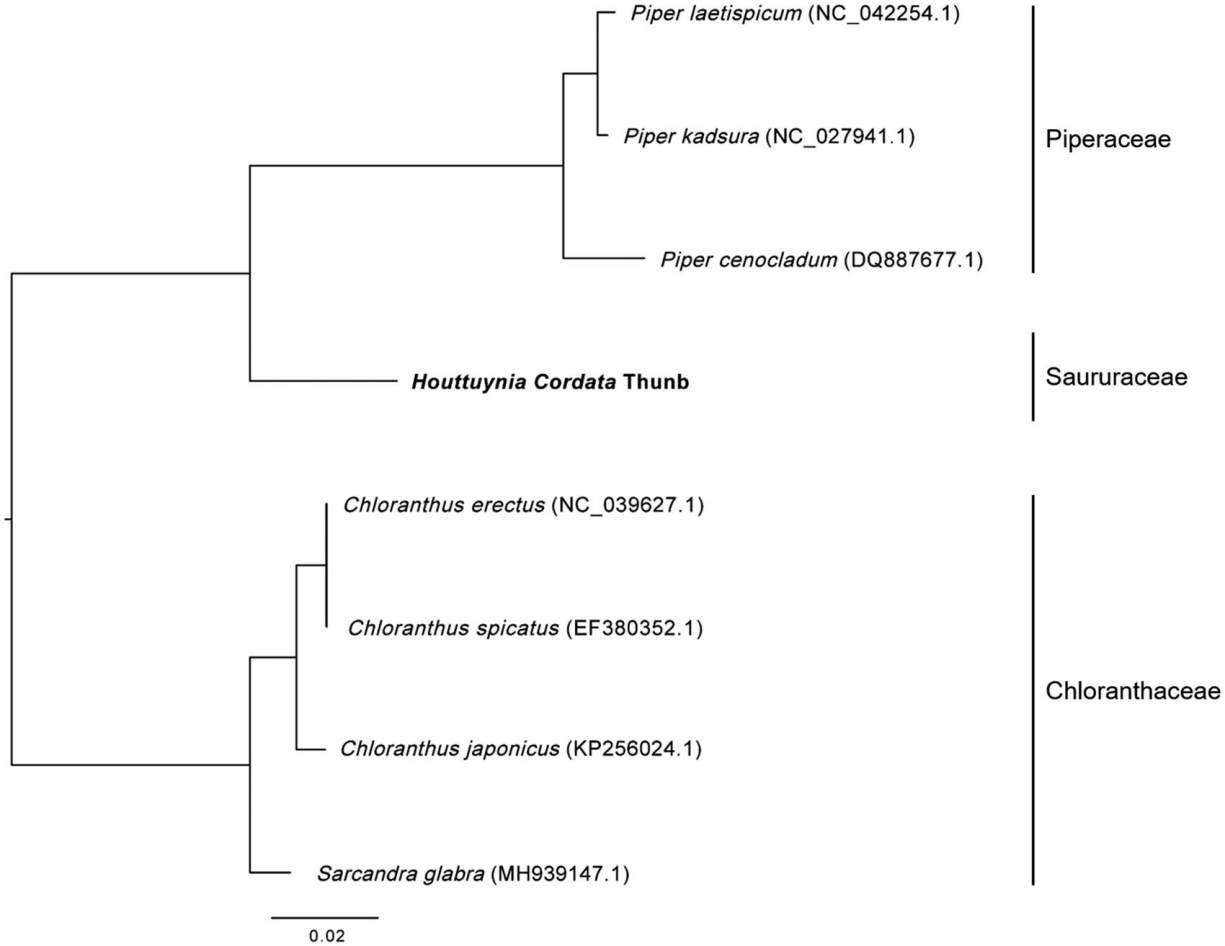
Maximum likelihood phylogenetic tree based on the complete chloroplast genome sequences of plant species from Piperales.
